# An Insight into the Combined Toxicity of 3,4-Dichloroaniline with Two-Dimensional Nanomaterials: From Classical Mixture Theory to Structure-Activity Relationship

**DOI:** 10.3390/ijms24043723

**Published:** 2023-02-13

**Authors:** Zhuang Wang, Le Yu

**Affiliations:** School of Environmental Science and Engineering, Collaborative Innovation Center of Atmospheric Environment and Equipment Technology, Jiangsu Key Laboratory of Atmospheric Environment Monitoring and Pollution Control, Nanjing University of Information Science and Technology, Nanjing 210044, China

**Keywords:** multifunctional nanomaterials, aquatic toxicity, structure-activity relationship, *in silico*, combined pollution

## Abstract

The assessment and prediction of the toxicity of engineered nanomaterials (NMs) present in mixtures is a challenging research issue. Herein, the toxicity of three advanced two-dimensional nanomaterials (TDNMs), in combination with an organic chemical (3,4-dichloroaniline, DCA) to two freshwater microalgae (*Scenedesmus obliquus* and *Chlorella pyrenoidosa*), was assessed and predicted not only from classical mixture theory but also from structure-activity relationships. The TDNMs included two layered double hydroxides (Mg-Al-LDH and Zn-Al-LDH) and a graphene nanoplatelet (GNP). The toxicity of DCA varied with the type and concentration of TDNMs, as well as the species. The combination of DCA and TDNMs exhibited additive, antagonistic, and synergistic effects. There is a linear relationship between the different levels (10, 50, and 90%) of effect concentrations and a Freundlich adsorption coefficient (*K*_F_) calculated by isotherm models and adsorption energy (*E*_a_) obtained in molecular simulations, respectively. The prediction model incorporating both parameters *K*_F_ and *E*_a_ had a higher predictive power for the combined toxicity than the classical mixture model. Our findings provide new insights for the development of strategies aimed at evaluating the ecotoxicological risk of NMs towards combined pollution situations.

## 1. Introduction

Along with the rapid development of nanotechnology, various structural and functionalized nanomaterials (NMs) have emerged and are widely used in human society [1,2]. Nanotechnology is changing our lives, while engineered nanoparticles (ENPs) have become an environmental pollutant [3]. The reality of pollution in the natural or artificial environment is that it is mostly a combined pollution. This also means that ENPs rarely exist alone but enter the environment at the same time as, or in sequence with, other pollutants and interact with each other to form combined pollution [4]. It is undeniable that the combined effect of ENPs with other pollutants on organisms is a worthwhile concern.

To address the problem of combined pollution in the presence of ENPs, a series of relevant experimental and theoretical research works have been carried out [5,6]. Some studies have shown that the presence of ENPs increases the biological impact of other contaminants [7,8]; others have shown that the presence of ENPs decreases the biological impact of other contaminants [9,10]. The main reason for this difference in the types of interaction may be related to the differences in the types of NMs and species. The development of methods for predicting the mixture toxicity of NMs is lagging relatively behind [11]. Previous studies have reported that classical mixture models are effective in assessing the toxicity of mixtures of ENPs [12,13,14]. Recently, parametric predictive modelling approaches such as quantitative structure-activity relationships (QSAR) have been gradually applied to the prediction of the toxicity of mixtures of ENPs [15,16]. One of the key issues in developing effective parametric prediction models is the search for universal descriptors. However, the descriptors in the existing models developed are dominated by a selection of physicochemical properties of the NMs themselves, with little consideration given to descriptors that could describe the interactions between the mixed components.

Due to their superior adsorption ability, two-dimensional NMs (TDNMs) are widely used for environmental pollutant removal [17,18]. This provides the opportunity for TDNMs to co-exist with other pollutants. Amongst TDNMs, two advanced NMs, namely layered double hydroxides (LDHs) [19] and graphenes [20], show their strengths. This study aimed to investigate the toxicity of binary mixtures of TDNMs and an organic chemical on aquatic organisms and to assess the joint effects, as well as to make quantitative predictions of the combined toxicity. 3,4-Dichloroaniline (DCA) was selected as the model compound and it is an important intermediate for a variety of pesticides, pharmaceuticals, dyes, pigments, and fine chemicals, and is also a bioactive intermediate [21]. Moreover, DCA is one of the most widely produced anilines worldwide and has been detected in freshwater, brackish, and marine environments [22]. Two freshwater microalgae (*Scenedesmus obliquus* [23] and *Chlorella pyrenoidosa* [24]) which have been frequently used to monitor the toxicity of NMs were chosen as test organisms. Two widely used LDHs (Mg-Al-LDH [25] and Zn-Al-LDH [26]) and a typical graphene nanoplatelet (GNP) [27] were selected as the model TDNMs. A classical mixture toxicity model and a developed parametric prediction model were applied to assess and predict the combined toxicity of TDNMs and DCA to the microalgae. This study pioneered the development of quantitative predictions of the combined toxicity between TDNMs and organic pollutants.

## 2. Results and Discussion

### 2.1. Combined Toxicity of DCA with TDNMs

**Figure 1:**
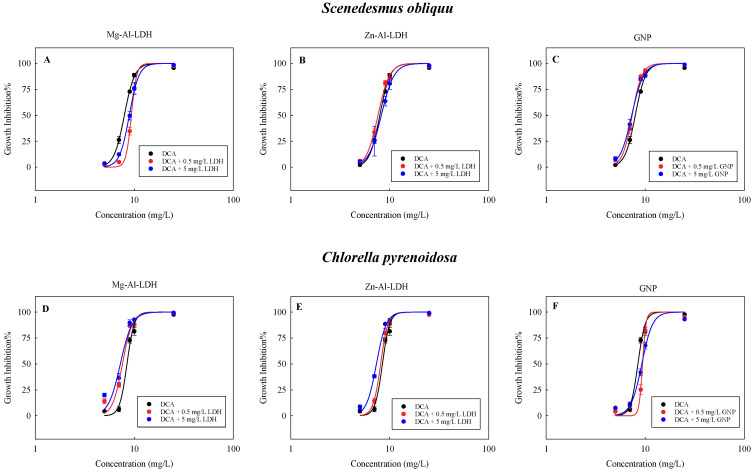
Concentration-response curves for the growth inhibition of the two freshwater microalgae exposed to the DCA in the absence and presence of Mg-Al-LDH (**A**,**D**), Zn-Al-LDH (**B**,**E**), and GNP (**C**,**F**). Data are mean ± SD (*n* = 3).

Figure 1 presents the growth inhibition toxicity of DCA to *S. obliquu* and *C. pyrenoidosa* in the absence and presence of Mg-Al-LDH, Zn-Al-LDH, or GNP. Generally, with increasing the concentration of DCA, the rate of growth inhibition of the two algae increased, implying that the DCA had a growth inhibition effect on the algae in a concentration-dependent manner. In the presence of the three TDNMs, the DCA also had a concentration-response relationship with the algae. However, the CRCs for the DCA in the presence of TDNMs deviated from the CRCs for the DCA alone to varying degrees. For *S. obliquu*, the presence of Mg-Al-LDH shifted the CRC of DCA in the direction of less toxicity (Figure 1A). Conversely, the presence of GNP shifted the CRC of DCA in the direction of greater toxicity (Figure 1C). Unlike all other TDNMs, the presence of 0.5 mg/L Zn-Al-LDH shifted the CRC of DCA in the direction of greater toxicity, while 5 mg/L Zn-Al-LDH shifted the CRC of DCA in the direction of less toxicity (Figure 1B).

For *C. pyrenoidosa*, as the concentrations of Mg-Al-LDH (Figure 1D) and Zn-Al-LDH (Figure 1E) increased, the CRCs of DCA moved in the direction of increased toxicity, and the higher the concentration of LDHs, the more pronounced was the tendency for the CRCs of DCA to move in the direction of increased toxicity. Unlike the LDHs, the GNP shifted the CRC of DCA in the direction of reduced toxicity at different concentrations (Figure 1F). Taken together, the extent of the deviation of CRCs for DCA was also related to the type and concentration of TDNMs, as well as the type of species.

To quantify the toxicity of DCA to both algae, we determined the *EC*_10_, *EC*_50_, and *EC*_90_ values of DCA in the presence of NMs using the CRCs curves (Table 1). For *S. obliquu*, the average *EC*_X_ values of DCA when the Mg-Al-LDH was present were greater than the DCA alone, implying that the presence of Mg-Al-LDH decreased the toxicity of DCA. On the contrary, the presence of Zn-Al-LDH and GNP enhanced the toxicity to *S. obliquu*. Moreover, the lower the concentration of Zn-Al-LDH, the greater the increase in the DCA toxicity. Unlike the Zn-Al-LDH, in terms of *EC*_10_ and *EC*_50_, the DCA became more toxic as the GNP concentration increased. For *C. pyrenoidosa*, the average *EC*_X_ values of DCA when the Mg-Al-LDH or Zn-Al-LDH was present were lower than the DCA alone (Table 1), implying that the presence of Mg-Al-LDH or Zn-Al-LDH increased the toxicity of DCA. Moreover, the toxicity of DCA increased with increasing the LDH concentration. Unlike the Mg-Al-LDH and Zn-Al-LDH, GNP mitigated the toxicity of DCA to *C. pyrenoidosa*.

### 2.2. Types of Joint Toxic Action of DCA and TDNMs

**Figure 2:**
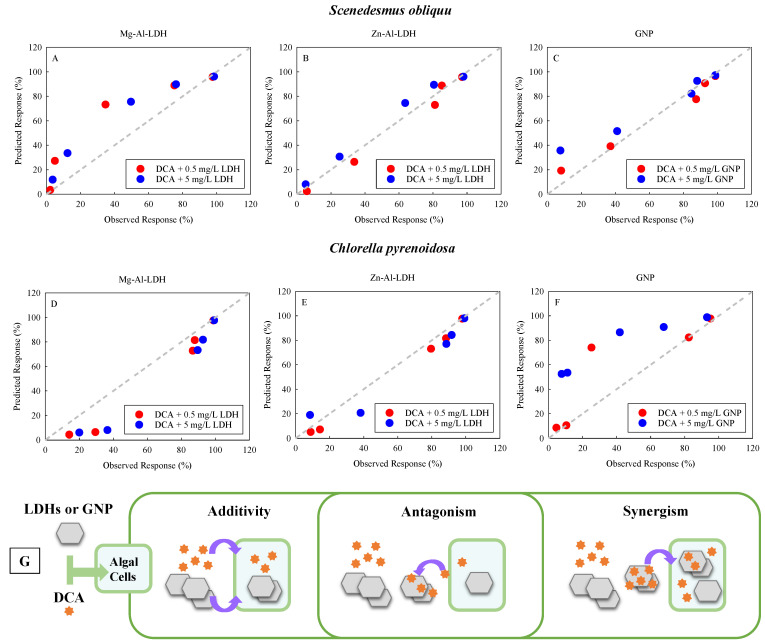
Summary of the experimentally determined (observed) and predicted toxicity response of DCA in the absence and presence of Mg-Al-LDH (**A**,**D**), Zn-Al-LDH (**B**,**E**), and GNP (**C**,**F**). Schematic diagram (**G**) shows the types of joint interactions between the studied ENPs and DCA towards algal cells.

Figure 2 shows the observed and predicted values of the combined toxic response of DCA with the three TDNMs to *S. obliquus* and *C. pyrenoidosa*, respectively. As shown in Figure 2, the dotted line represents perfect agreement between the observed and predicted values. For *S. obliquus*, the values of response for the combined DCA and Mg-Al-LDH were in the region where the predicted values were greater than the observed values (Figure 2A), indicating that the joint interaction between the DCA and Mg-Al-LDH was antagonistic. Noticeably, when the concentrations of DCA were at their lowest and highest, the observed joint response was almost comparable to the predicted joint response, implying an additive mode of joint toxic action of DCA and Mg-Al-LDH. For the binary combination of DCA and Zn-Al-LDH, the observed and predicted response were in a good agreement (*R* = 0.985), suggesting that the joint toxic action of DCA and Zn-Al-LDH was additive (Figure 2B). As shown in Figure 2C, when the DCA was at its lowest concentration, the DCA showed an antagonistic effect with GNP as the observed response was lower than the predicted response. Otherwise, the observed response for the joint exposure of DCA and GNP was almost equal to the predicted response, indicating that the joint effect of DCA and GNP was in an additive manner.

For *C. pyrenoidosa*, with the exception of the response point corresponding to the DCA at the highest concentration in the presence of Mg-Al-LDH, the predicted values were lower than the observed values (Figure 2D), indicating that the joint interaction between the DCA and Mg-Al-LDH was synergistic. The joint toxic action of DCA at the highest concentration and Mg-Al-LDH was additive. As shown in Figure 2E, the observed and predicted response were in a good agreement (*R* = 0.982), suggesting that the joint toxic action of DCA and Zn-Al-LDH was additive. The predicted response corresponding to the DCA at a moderate concentration in the presence of GNP was higher than the observed response (Figure 2F), implying that the joint interaction between the DCA and 0.5 mg/L GNP was antagonistic. Except for this, the observed response was almost equal to the predicted response, suggesting that the joint toxic action of DCA and 0.5 mg/L GNP was additive. The values of response for the combined DCA and 5 mg/L GNP was in the region where the predicted values were greater than the observed values (Figure 2F), indicating that the joint interaction between the DCA and 5 mg/L GNP was antagonistic.

To sum up, the joint effects of DCA and TDNMs on the freshwater algae are mainly additive, antagonistic, and synergistic, which are related to the type of species and the concentration of the mixed components. Previous studies also show joint toxic action with different types in the same mixture of ENPs and co-existing contaminants depending on test species [4] and the exposure levels of test materials [28].

Figure 2G depicts a schematic diagram of the different types of joint toxic action. The combined toxicity of DCA and TDNMs works in an additive manner in the sense that the toxicity of a mixture of DCA and TDNMs is equal to the sum of the toxicity of each mixture component acting alone. Previous studies also stated that treatments of both LDHs and GNP alone caused adverse impacts on microalgae [29,30]. Antagonistic combined toxicity effects of DCA and TDNMs were shown under thee lower bioavailable DCA as competitive adsorption of DCA by TDNMs and algae cells. With the TDNMs presented, the toxicity of DCA to the algae could be reduced. On the contrary, synergistic combined toxicity effects were induced via mechanical and/or oxidative damages by TDNMs and the promoted delivery of DCA into the algal cells by the TDNMs as carriers, i.e., the Trojan Horse phenomenon [31]. More specifically, the TDNMs might deliver more DCA into the algal cell through the Trojan Horse effect and release the DCA within the cell. This interaction might increase the amount of DCA that reached the algal cells and could synergistically generate intracellular reactive oxygen species (ROS) by the combination, thereby worsening the oxidative stress effect on the algal cells.

### 2.3. Correlation between Interaction Parameters and Combined Toxicity

To further investigate the impacts of the adsorption on the joint toxicities of the DCA and the TDNMs, the Freundlich isotherm fitting parameters and simulated *E*_a_ for the adsorption of the DCA on the TDNMs were determined (Table 2). As shown in Table 2, the goodness-of-fit (*R*^2^) of the model to the experimental data indicated that the Freundlich model fitted the isotherms for the adsorption of the DCA on the TDNMs well. The 1/*n* values implied that the DCA preferred to adsorb to the surface of TDNMs. Some previous studies have also demonstrated the adsorption of organic chemicals to LDHs [32] or graphene-based materials [18]. It was also found that the Freundlich adsorption coefficient (*K*_F_) decreased in the order Mg-Al-LDH > Zn-Al-LDH > GNP. Moreover, the absolute *E*_a_ decreased in the order Mg-Al-LDH > Zn-Al-LDH > GNP, implying that the Mg-Al-LDH showed the strongest adsorption capacity for the DCA, while the GNP showed the weakest adsorption capacity for the DCA. Furthermore, as the concentration of TDNMs increased, the value of *K*_F_ became smaller and 1/*n* became larger (Table 2), namely that the adsorption isotherm decreased with increasing the adsorbent concentration. Possible explanations for this include the fact that ENPs are prone to agglomeration in the aqueous phase, which affects the dispersion of the particles. Moreover, the concentration of ENPs can influence both their agglomeration and dispersion [13]. Furthermore, the formation of agglomerates of ENPs may change their physicochemical properties, which in turn may change the surface effect, thus reducing the adsorption capacity of NMs. It can be also concluded that there was an adsorbent concentration effect. Several current investigations on the adsorption performance of various materials have also revealed the effect of adsorbent concentration [33,34,35].

**Figure 3:**
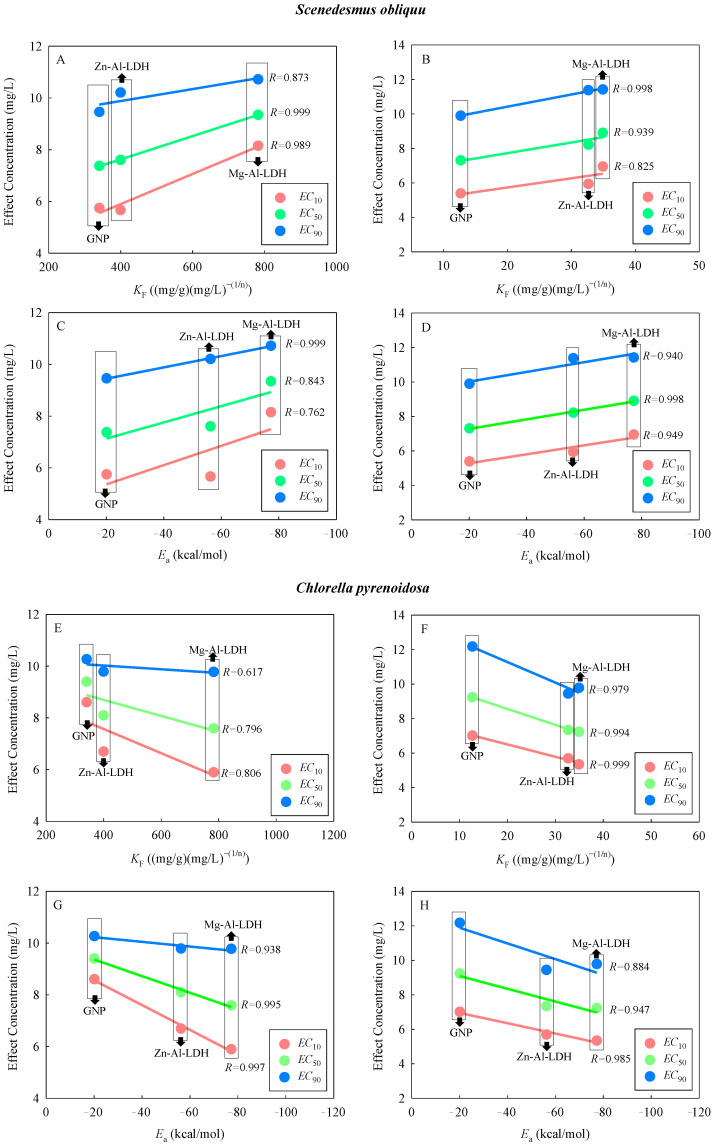
Variation of different effect concentrations (*EC*_X_, X = 10, 50, and 90) of DCA in the presence of 0.5 (**A**,**C**,**E**,**G**) and 5 (**B**,**D**,**F**,**H**) mg/L of Mg-Al-LDH, Zn-Al-LDH, and GNP with the Freundlich isotherm fitting parameter (*K*_F_) (**A**,**B**,**E**,**F**) and simulated adsorption energies (*E*_a_) (**C**,**D**,**G**,**H**).

Furthermore, the relationship between the effect concentrations (*EC*_10_, *EC*_50_, and *EC*_90_) and *K*_F_ or *E*_a_ was conducted (Figure 3). For *S. obliquus*, the selected effect concentrations were positively correlated with the *K*_F_ and absolute *E*_a_ values. This implies that the joint toxicities decreased with the increase in the adsorption capability of the TDNMs. This suggests that the greater the amount of the isolated DCA in the binary mixture systems, the higher the joint toxicity of the mixture. However, for *C. pyrenoidosa*, negative correlations were found between the effect concentrations values and the *K*_F_ and absolute *E*_a_ values, suggesting that the joint toxicities increased with the increase in the adsorption capability of the TDNMs. In a sense, on the other hand, it means that the adsorbed DCA contributed more to the joint toxicities.

### 2.4. Prediction of Combined Toxicity of DCA and TDNMs

**Figure 4:**
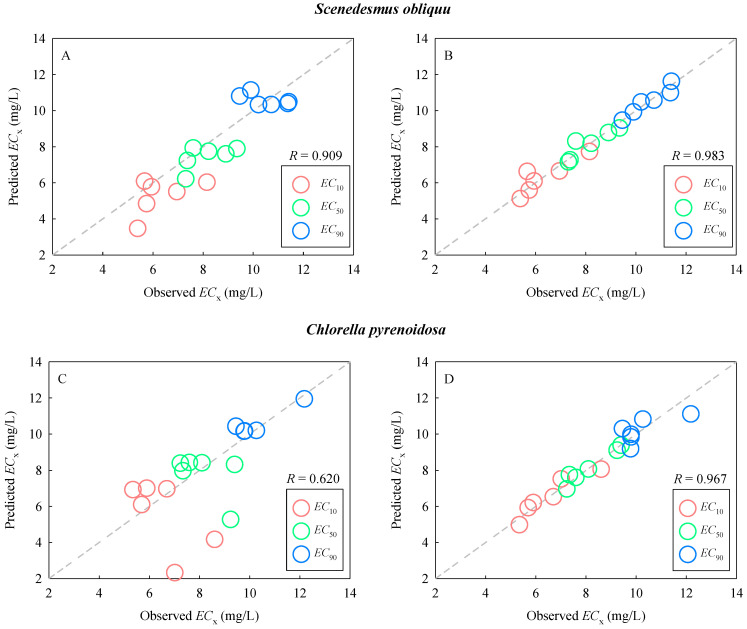
Plot of experimentally determined (observed) values of effect concentrations (*EC*_X_, X = 10, 50, and 90) versus predicted values by the Abbott model (**A**,**C**) and parametric prediction models (**B**,**D**). The dashed line represents perfect agreement between experimental and calculated values.

The Abbott model was used to predict the combined toxicity of DCA with TDNMs to *S. obliquus* and *C. pyrenoidosa*. A plot of experimentally observed versus predicted *EC*_X_ (X = 10, 50, and 90) is presented in Figure 4A and Figure 4C, respectively. The dotted line represents perfect agreement between observed and predicted values. The agreement (*R* = 0.909) between the observed effect concentration values and those predicted by the Abbott model is satisfactory for *S. obliquus*. However, the values predicted by the Abbott model showed a moderate correlation (*R* = 0.620) with the observed values for *C. pyrenoidosa*. The predictive power of the classical mixture model was related to the choice of species tested. This also reveals a distinct sensitivity of the two algae to the mixture of DCA and TDNMs.

As aforementioned, the toxic effect concentrations were *K*_F_- and *E*_a_-dependent (Figure 3). On the basis of this, we developed parametric prediction models incorporating the two parameters (Table 3). A plot of experimentally observed versus predicted *EC*_X_ (X = 10, 50, and 90) is presented in Figure 4B for *S. obliquus* and Figure 4D for *C. pyrenoidosa*, respectively. The agreement between the observed effect concentration values and those predicted by the parametric prediction models is satisfactory for both *S. obliquus* (*R* = 0.983) and *C. pyrenoidosa* (*R* = 0.967). A comparative analysis revealed that the parametric prediction models performed better than the Abbott model. The classical mixture models often rely on the determination of concentration-response relationships for single components. In particular, the most common conceptual models, such as concentration addition and independent action, require prior knowledge of the modes of action of the components on species [36,37]. In contrast to these classical mixture models, descriptors in parametric predictive models are easily available and do not depend on the test species. In addition, for both *S. obliquus* and *C. pyrenoidosa*, the parametric prediction models constructed for the *EC*_50_ values showed good robustness and internal predictability (Q^2^_CUM_ > 0.5, Q^2^_CUM_ is the cumulative percentage of variance explained for extracted components), and the models thus have high goodness-of-fit (Table 3).

As mentioned above, the concentration of ENPs has an impact on their aqueous agglomeration and dispersion. From the industrial production point of view, a positive implication of this study is to obtain the range of variation of the adsorption performance of ENPs in the aqueous phase dependent on the ENP concentrations, which provides a reference for the development and manufacturing of novel NMs. This study may also pave the way to improve the adsorption performance of NMs for other contaminants in the aquatic environment, such as designing hydrophilic nanoparticles by a surface modification to ensure good dispersion in an aqueous environment, which in turn improves the adsorption performance of NMs.

The new insight proposed in this study provides a methodological basis for accelerating the ecological risk assessment of mixtures of NMs and multifunctional NMs as well as a theoretical basis for the design and production of green and low-toxic NMs. In the face of numerous types of environmental pollutants and the continuous emergence of new NMs, further research requires the expansion of data sets, as well as the application of big data, artificial intelligence, and machine learning to establish assessment and prediction models for the toxicity of NMs mixed with other environmental pollutants.

## 3. Materials and Methods

### 3.1. Test Materials, Test Species, and Test Medium

The selected Mg-Al-LDH and Zn-Al-LDH with a diameter of 1–4 μm were purchased from Nanjing XFNANO Materials Tech Co., Ltd. (Nanjing, China). GNP was purchased as powders with a thickness of 1–4 nm and a particle size of 2 μm from PlasmaChem GmbH (Berlin, Germany). A stock suspension with a concentration of 5 g/L of the test NMs was prepared using pure water as a solvent, sonicated (150 W, 40 kHz, 25 °C, 30 min) and magnetically stirred for 1 h to disperse the materials before use. DCA (CAS 95-76-1, 98% purity) was purchased from Aladdin Industrial Co. (Shanghai, China). The unicellular freshwater algae *S. obliquus* and *C. pyrenoidosa* were obtained from the Chinese Academy of Sciences Institute of Hydrobiology (Wuhan, China). The test medium (pH 7.8 ± 0.2) was freshly prepared according to the OECD technical guideline 201 [38].

### 3.2. Bioassay

Exponentially growing algae cells (with a final density of 3 × 10^5^ cells/mL for *S. obliquus* and 4 × 10^5^ cells/mL for *C. pyrenoidosa*) were added to the control and treated experiments. All flasks containing various treatments were incubated in an artificial growth chamber at a consistent temperature of 24 ± 1 °C with a photoperiod of 12 h light (3000–4000 lx) and 12 h dark. The algal cell density was determined using an ultraviolet-visible spectrophotometer (UV1102; Shanghai Tian Mei Scientific Instrument Co., Shanghai, China) after 96 h for *S. obliquus* and 72 h for *C. pyrenoidosa* to provide cell numbers and allow the specific growth inhibition rate to be calculated.

### 3.3. Concentration-Response Relationship

Effect concentrations (i.e., *EC*_10_, *EC*_50_, and *EC*_90_) of DCA in the absence and presence of LDHs and GNP were determined by concentration-response curves (CRCs). CRCs were constructed by a nonlinear logistic regression model:

(1) $$E=\frac{100}{\left(1+{\left(\frac{C}{EC_{50}}\right)}^{\rho }\right)}$$

where *E* is the effect confined to the range 0–100%, *C* is the exposure concentration of the test materials, *EC*_50_ is the median effect concentration, and *ρ* represents the slope of CRC.

### 3.4. Assessment and Prediction of Combined Toxicity

The Abbott model was applied to determine the types of interactions between DCA and TDNMs when they coexist as a binary mixture [39]. The joint toxic action of DCA and TDNMs can be estimated by comparing the observed toxicity response of the binary mixtures of DCA and TDNMs (*TR*_Obs_) with the predicted toxicity response (*TR*_Pre_), as shown in Equation (2):

(2) $$TR_{Pre}=TR_{\mathrm{DCA}}+TR_{\mathrm{TDNMs}}-(TR_{\mathrm{DCA}}\cdot TR_{\mathrm{TDNMs}}/100)$$

If *R*_Obs_ was noticeably higher or lower than *R*_Pre_, respectively, the result estimated was utilized to signify a synergistic or antagonistic effect. On the contrary, the interaction of the binary mixture was considered an additive effect only if there was no significant difference between *R*_Obs_ and *R*_Pre_. Furthermore, the same method was used to predict the toxicity of the binary mixtures of DCA and TDNMs.

### 3.5. Adsorption Experiments and Molecular Simulation

The binary mixtures of DCA and TDNMs (pH = 7.8 ± 0.2) for adsorption experiments were equilibrated by stirring for 30 min at 25 °C in a dark room. Afterwards, each test sample was passed through a syringe filter with 0.02 μm pore diameter (Antop 25, Whatman) and the corresponding control experiments were paired. The equilibrium DCA concentrations in the samples were determined using a series of standard curves (*R*^2^ = 0.9997). The Freundlich model Equation (3) was fitted to the sorption isotherm.

(3) $$q_{e}=K_{F}C_{e}{}^{\frac{1}{n}}$$

where *q*_e_ is the equilibrium adsorption capacity (mg/g), *C*_e_ is the equilibrated concentration (mg/L), *K*_F_ is the Freundlich adsorption coefficient ((mg/g)(mg/L)^−(1/*n*)^), and 1/*n* is the Freundlich intensity parameter.

The molecular structures of LDHs and graphene materials were constructed according to the proposed models described in previous studies [40,41]. Calculations were performed for isolated molecules and complexes using the molecular mechanics (MM) simulation [42]. For the interaction systems, adsorption energy (*E*_a_) was used to evaluate the stability of the complexes of the TDNMs with DCA. The magnitude of *E*_a_ is an indication of the magnitude of the driving force towards complexation. A negative value reflects a stable adsorption of DCA on the TDNMs. *E*_a_ was calculated by

(4) $$E_{a}=E_{\text{TDNM-DCA}}-E_{\mathrm{TDNM}}-E_{\mathrm{DCA}}$$

where *E*_TDNM-DCA_, *E*_TDNM_, and *E*_DCA_ represent the total energies of the complex, the isolated TDNMs, and the individual DCA, respectively. The total energies were determined by minimizing the molecular structures using the smart geometry optimization algorithms. The universal force field was adopted to perform this simulation. The cutoff radius was chosen to be 18.5 Å.

### 3.6. Development of a Parametric Prediction Model for EC_x_

Based on the interactions between the TDNMs and the DCA implied by the MM simulations, *K*_F_ and *E*_a_ were selected to correlate with *EC*_x_ and an orthogonal partial least squares regression was performed with Simca (Ver. 14.1 Umetri AB and Erisoft AB) to develop predictive models.

## 4. Conclusions

In summary, an analysis of the combined toxicity of DCA with TDNMs to the algae *S. obliquus* and *C. pyrenoidosa* was undertaken. The joint exposure assessment revealed additive, antagonistic and synergistic effects. The prediction model integrating the two descriptors (*K*_F_ and *E*_a_) based on the notion of QSAR provided a valid prediction of the combined toxicity and performed comparably better than the classical mixture model. The parametric methodology chosen for the present study appears to be suitable for the assessment of the toxicity of multicomponent NMs.

## Figures and Tables

**Table 1:** Toxic effect concentrations of the 3,4-dichloroaniline (DCA) in the absence and presence of 0.5 and 5 mg/L of Mg-Al-LDH, Zn-Al-LDH, and GNP *^a^*.

Studied Systems	Effect Concentrations (mg/L)					
	*EC*_ 10 _		*EC*_ 50 _		*EC*_ 90 _	
	*S. obliquu*	*C. pyrenoidosa*	*S. obliquu*	*C. pyrenoidosa*	*S. obliquu*	*C. pyrenoidosa*
DCA	6.10 [6.04–6.14]	7.01 [6.84–7.07]	7.94 [7.85–8.03]	8.44 [8.25–8.62]	10.33 [10.03–10.67]	10.16 [9.63–10.87]
+Mg-Al-LDH (0.5)	8.15 [8.05–8.22]	5.90 [5.64–5.98]	9.35 [9.29–9.41]	7.59 [7.34–7.84]	10.72 [10.50–10.99]	9.78 [9.01–10.91]
+Mg-Al-LDH (5)	6.95 [6.81–7.05]	5.34 [4.92–5.46]	8.91 [8.81–9.00]	7.23 [6.90–7.57]	11.42 [11.02–11.90]	9.79 [8.72–11.64]
+Zn-Al-LDH (0.5)	5.67 [5.57–5.73]	6.70 [6.59–6.73]	7.61 [7.49–7.72]	8.10 [7.92–8.27]	10.21 [9.80–10.70]	9.79 [9.33–10.39]
+Zn-Al-LDH (5)	5.94 [5.87–5.99]	5.69 [5.58–5.76]	8.22 [8.15–8.29]	7.34 [7.22–7.45]	11.38 [11.08–11.70]	9.45 [9.05–9.94]
+GNP (0.5)	5.75 [5.65–5.81]	8.61 [8.48–8.65]	7.37 [7.27–7.48]	9.40 [9.29–9.51]	9.46 [9.10–9.89]	10.27 [9.98–10.68]
+GNP (5)	5.39 [5.32–5.44]	7.01 [6.56–7.24]	7.31 [7.22–7.31]	9.24 [9.04–9.44]	9.90 [9.57–10.29]	12.18 [11.29–13.59]

*^a^* The two-sided confidence intervals are given in brackets.

**Table 2:** Freundlich isotherm fitting parameters and simulated adsorption energies for the adsorption of the 3,4-dichloroaniline (DCA) on 0.5 and 5 mg/L of Mg-Al-LDH, Zn-Al-LDH, and GNP.

Studied Systems	Isotherm-Freundlich			Complex Configuration	Simulated Adsorption Energies
	*K*_ F _ (mg/g)(mg/L) ^ − (1/*n*) ^	1/*n*	*R* ^2^		*E*_ a _ (kcal/mol)
Mg-Al-LDH (0.5)	782.35	0.35	0.91	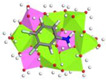	−77.26
Mg-Al-LDH (5)	34.92	0.79	0.99		
Zn-Al-LDH (0.5)	399.67	0.46	0.88	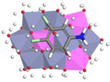	−56.24
Zn-Al-LDH (5)	32.67	0.90	0.99		
GNP (0.5)	340.80	0.31	0.96	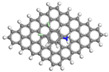	−20.11
GNP (5)	12.68	0.50	0.84		

**Table 3:** The developed parametric prediction models and their performance *^a^*.

*S. obliquu*	
Model 1	*EC*_10_ = 6.005 + 0.418 · *K*_F_ − 0.645 · *E*_a_ *N* = 6, *R*^2^ = 0.762, RMSE = 0.683, Q^2^_CUM_ = 0.493
Model 2	*EC*_50_ = 9.552 + 0.121 · *K*_F_ − 0.861 · *E*_a_ *N* = 6, *R*^2^ = 0. 822, RMSE = 0.473, Q^2^_CUM_ = 0.629
Model 3	*EC*_90_ = 9.339 − 0.001 · *K*_F_ − 0.030 · *E*_a_ *N* = 6, *R*^2^ = 0.911, RMSE = 0.310, Q^2^_CUM_ = 0.819
*C. pyrenoidosa*	
Model 4	*EC*_10_ = 5.500 + 0.421 · *K*_F_ + 0.981 · *E*_a_ *N* = 6, *R*^2^ = 0.877, RMSE = 0.660, Q^2^_CUM_ = 0.630
Model 5	*EC*_50_ = 8.528 − 0.272 · *K*_F_ + 1.022 · *E*_a_ *N* = 6, *R*^2^ = 0.941, RMSE = 0.302, Q^2^_CUM_ = 0.880
Model 6	*EC*_90_ = 10.210 − 0.264 · *K*_F_ + 0.568 · *E*_a_ *N* = 6, *R*^2^ = 0.487, RMSE = 0.865, Q^2^_CUM_ = 0.102

*^a^ N* stands for the number of mixtures, *R*^2^ is squared regression coefficient, RMSE is root mean squared error, and Q^2^_CUM_ is the cumulative percentage of variance explained for extracted components.

## Data Availability

Data presented in this study are available on request from the corresponding author.
